# Tag SNPs detect association of the *CYP1B1* gene with primary open angle glaucoma

**Published:** 2010-11-04

**Authors:** Kathryn P. Burdon, Alex W. Hewitt, David A. Mackey, Paul Mitchell, Jamie E. Craig

**Affiliations:** 1 Department of Ophthalmology, Flinders University, Adelaide, Australia; 2 Centre for Eye Research Australia, University of Melbourne, Royal Victorian Eye and Ear Hospital, Melbourne, Australia; 3 Centre for Vision Research, Department of Ophthalmology and Westmead Millennium Institute, University of Sydney, Westmead, Australia

## Abstract

**Purpose:**

The cytochrome p450 family 1 subfamily B (*CYP1B1*) gene is a well known cause of autosomal recessive primary congenital glaucoma. It has also been postulated as a modifier of disease severity in primary open angle glaucoma (POAG), particularly in juvenile onset families. However, the role of common variation in the gene in relation to POAG has not been thoroughly explored.

**Methods:**

Seven tag single nucleotide polymorphisms (SNPs), including two coding variants (L432V and N543S), were genotyped in 860 POAG cases and 898 examined normal controls. Each SNP and haplotype was assessed for association with disease. In addition, a subset of 396 severe cases and 452 elderly controls were analyzed separately.

**Results:**

There was no association of any individual SNP in the full data set. Two SNPs (rs162562 and rs10916) were nominally associated under a dominant model in the severe cases (p<0.05). A common haplotype (AGCAGCC) was also found to be nominally associated in both the full data set (p=0.048, OR [95%CI]=0.83 [0.69–0.90]) and more significantly in the severe cases (p=0.004, OR [95%CI]=0.68 [0.52–0.89]) which survives correction for multiple testing.

**Conclusions:**

Although no major effect of common variation at the *CYP1B1* locus on POAG was found, there could be an effect of SNPs tagged by rs162562 and represented on the AGCAGCC haplotype.

## Introduction

The cytochrome p450 family 1 subfamily B (CYP1B1) is a member of the CYP450 superfamily. While its exact function and effect on cells is not clear, the gene is inducible by dioxins and has several endogenous substrates including 17β-estradiol, retinoic acid, and melatonin as well as many exongenous substrates [1]. It was first recognized as a cause of primary congenital glaucoma (PCG) following linkage mapping [2] and candidate gene screening [3] in a panel of 17 Turkish families with recessive PCG. This finding has since been replicated in many ethnic groups with over 80 mutations now reported from many different populations [1,4,5]. In many cases, compound heterozygosity is observed as the cause of recessive disease. The proportion of PCG cases accounted for by *CYP1B1* mutations varies significantly between ethnic groups, from around 20% in Australia and Japan to nearly 100% in Saudi Arabia and Slovakian Gypsies [4].

Primary Open Angle Glaucoma (POAG) is the most prevalent form of glaucoma and leads to significant levels of irreversible blindness worldwide. The genetics of this complex trait are not well understood, although many loci and several genes have been reported [6]. The most common known genetic cause of POAG is the myocilin gene (*MYOC*). Mutations in this gene account for 2%–4% of POAG in Caucasians [7] and up to 36% in juvenile onset (JOAG) families [8]. *CYP1B1* mutations have also been identified in JOAG and POAG patients. Melki et al. [9] reported compound heterozygotes in three French families containing patients with PCG as well as JOAG. Acharya et al. [10] reported nine individuals from India (4 JOAG and 5 POAG) with single heterozygous mutations in *CYP1B1* and Kumar et al. [11] reported four mutations in 27 Indian POAG patients, including two who were compound heterozygotes. Lopez-Garrido et al. [12] presented heterozygous mutations in 10 Spanish POAG patients. *CYP1B1* has also been suggested as a modifier of POAG in carriers of *MYOC* mutations [13]. A common polymorphism was associated with cupping of the optic disc, which may be relevant to POAG [14] although other studies found no association of *CYP1B1* mutations with disc changes in POAG [15].

Although several studies have reported rare variants in the *CYP1B1* gene in glaucoma patients that were not detected in controls [10-15], no large scale re-sequencing of normal population has been performed to determine the spectrum of rare variants in this gene. There are 75 reported coding variants in dbSNP, of which 45 are non-synonymous, 11 are insertions or deletions, and 4 are truncating mutations. While the majority have not yet been thoroughly validated as common polymorphisms, the number of reported frameshift and non-synonymous variants suggests that CYP1B1 activity is not compromised by most mutational events, at least in the heterozygous state. Thus, the presence of rare variants in the sequenced glaucoma patients is not surprising. The link between *CYP1B1* and POAG is therefore currently circumstantial. Chakrabarti et al. [15] assessed six common polymorphisims in a small cohort of POAG and primary angle closure glaucoma (PACG) patients as well as controls and found no association of any haplotypes with glaucoma status. This study aims to evaluate the contribution of common polymorphisms in *CYP1B1* to POAG.

## Methods

### Patients

Participants were drawn from the Glaucoma Inheritance Study in Tasmania (GIST), the Australian & New Zealand Registry of Advanced Glaucoma (ANZRAG) and the Blue Mountains Eye Study (BMES). The GIST and ANZRAG includes a clinic-based recruitment of glaucoma patients. The GIST aimed to capture all cases of glaucoma in Tasmania (an island state of Australia) and ANZRAG aims to capture cases of advanced glaucoma Australia-wide through ophthalmologist referral [16,17]. In both cases, normal elderly controls were ascertained from nursing home facilities in Launceston, Tasmania (for GIST) and Adelaide, South Australia (for ANZRAG). The BMES is a population based study of individuals aged over 50 years living in the Blue Mountains, west of Sydney, Australia [18]. All participants, including normal controls in all three studies were examined. Glaucoma was defined by concordant findings of typical glaucomatous visual field defects on the Humphrey 24–2 (for GIST and ANZRAG) or 30–2 (for BMES) test, together with corresponding optic disc rim thinning, including an enlarged cup-disc ratio (≥0.7) or cup-disc ratio asymmetry (≥0.2) between the two eyes. Intraocular pressure (IOP) was not considered in the diagnostic criteria. Advanced POAG was defined by a vertical cup:disc ratio >0.95, a best-corrected visual acuity worse than 6/60 due to POAG, or on a reliable Humphrey Visual Field (Carl Zeiss Pty. Ltd., Sydney, Australia) a mean deviation of ≤-22 db or at least 10 out of 16 central squares involved with a Pattern Standard Deviation of <0.5%. The field loss had to be due to POAG, and the less severely affected eye was required to have signs of glaucomatous disc damage and a glaucomatous field defect. Clinical exclusion criteria included: i) pseudoexfoliative glaucoma, ii) pigmentary glaucoma, iii) angle closure or mixed mechanism glaucoma; iv) secondary glaucoma due to aphakia, rubella, rubeosis or inflammation; v) congenital or infantile glaucoma, juvenile glaucoma with age of onset less than 20 years; or vi) glaucoma in the presence of a known syndrome.

All control subjects were required to have no known family history of POAG, as well as a normal intraocular pressure, optic disc and visual field. The population-based BMES control cohort comprised the eldest subgroup of people meeting control inclusion criteria.

### SNP selection and genotyping

Using the tagger program implemented in Haploview 4.0 [19] tag single nucleotide polymorphisms (SNPs) across the *CYP1B1* gene were selected on the basis of linkage disequilibrium patterns observed in the Caucasian (CEU) samples genotyped as part of the International HapMap Project [20]. Only SNPs with minor allele frequency greater than 5% in HapMap were considered. Two coding SNPs (rs1056836 and rs1800440 coding L432V and N453S respectively) and four 3′UTR SNPs (rs162549, rs2855358, rs10916, and rs162562) were force included to capture as much coding variation as possible. In addition, intronic SNPs rs10175368 and rs162556 were selected. These eight tag SNPs captured all alleles with an r^2^ of at least 0.8 (mean r^2^=0.96) and were gentoyped in all individuals using iPLEX GOLD chemsitry (Sequenom Inc., San Diego, CA) on an Autoflex Mass Spectrometer (Sequenom Inc.) at the Australian Genome Research Facility, Brisbane, Australia. The 3′UTR SNP rs2855658 failed genotyping and was removed from the analysis. This SNP did not tag any other HapMap SNPs.

### Statistical analysis

All analyses were conducted using the statistical genetics software packages Plink [21] and Haploview [19]. Hardy Wienberg equilibrium was assessed in all samples and in controls separately. Association was tested under the five genetic models implemented in Plink. These models are the allelic test (allele1 versus allele2), genotypic (11 versus 12 versus 22), dominant (11 and 12 versus 22), recessive (11 versus 12 and 22) and the Cochrane-Armitage Trend test. Association of common haplotypes (>1% frequency) was also assessed in Plink using the conditional haplotype test. All analyses were conducted in the full data set as well as a sub-set of cases with severe disease (from GIST and ANZRAG) compared to elderly (>81 years of age) examined controls. Power calculations [22] revealed that, assuming a prevalence of 3% in this population, for a sample of this size (860 cases versus 898 controls) under an additive model we had 99% power to detect a genotype relative risk of 1.1 for an allele frequency of 0.4 at α=0.007 (allowing for multiple testing of 7 SNPs). For an allele frequency of 0.2, the power is 71% for a relative risk of 1.1, 92% for 1.2, and 98% for 1.3.

## Results

In total, 860 cases and 897 examined, normal, unrelated controls were available. Sex and age distribution for the full cohort and each sub-cohort are given in Table 1. Overall, the age of cases is significantly less than the controls, although in the ANZRAG cohort the cases are slightly older. There were no differences in the proportion of each cohort that is female.

**Table 1:** Demographics by recruitment center.

** **	**N**		**Sex (% female)**			**Age (mean±SD)**		
**Cohort**	**Cases**	**Controls**	**Cases**	**Controls**	**p-value**	**Cases**	**Controls**	**p-value**
Full Sample	860	897	0.52	0.53	0.82	74.8	80.5	<0.001
Severe Sample	396	452	0.53	0.53	0.98	77.0	85.3	<0.001
ANZRAG	230	285	0.47	0.55	0.05	82.4	76.0	<0.001
GIST	476	101	0.60	0.68	0.16	73.3	86.3	<0.001
BMES	154	511	0.36	0.48	0.01	77.7	81.1	<0.001

**Figure 1:**
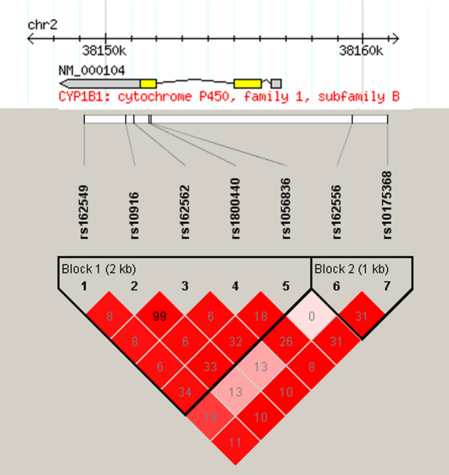
Linkage disequilibrium pattern between tag SNPs typed in the *CYP1B1* gene. R^2^ values are given in the intersecting boxes, with darker colors indicating stronger linkage disequilibrium. The position of each SNP relative to the gene is indicated. Figure generated in Haploview [19].

All seven SNPs were in Hardy–Weinberg equilibrium. Allele and genotype frequencies by glaucoma status are shown in Table 2. Linkage disequilibrium across the region is high with all seven SNPs falling into a single block, although the correlation between SNPs rs162556 and rs10175368 is low (Figure 1), consistent with values observed in the HapMap data set. Thus using the block definition of Gabriel et al. [23] there are two haplotype blocks as shown in Figure 1.

**Table 2:** Allele and genotype frequencies in all cases (n=860) and controls (n=897), presented as %(n).

** **	** **	** **	** **	** **	** **	** **	** **	**Genotype frequency**											
** **	** **	**Allele**		**Minor allele frequency**				**Cases**						**Controls**					
**SNP**	**Position (bp)**	**1**	**2**	**Cases**		**Controls**		**11**		**12**		**22**		**11**		**12**		**22**	
rs162549	38148960	T	A	0.24	(384)	0.23	(3946)	0.07	(53)	0.34	(278)	0.59	(478)	0.06	(48)	0.35	(298)	0.60	(509)
rs10916	38150674	G	T	0.21	(347)	0.23	(401)	0.05	(38)	0.32	(271)	0.63	(530)	0.06	(51)	0.34	(299)	0.60	(531)
rs162562	38151019	C	A	0.20	(346)	0.23	(404)	0.04	(37)	0.32	(272)	0.63	(536)	0.06	(51)	0.34	(302)	0.60	(537)
rs1800440 (N453S)	38151643	G	A	0.19	(313)	0.18	(318)	0.04	(34)	0.29	(245)	0.67	(566)	0.03	(28)	0.29	(262)	0.67	(600)
rs1056836 (L432V)	38151707	G	C	0.45	(739)	0.45	(788)	0.21	(168)	0.50	(403)	0.30	(243)	0.22	(188)	0.48	(412)	0.31	(267)
rs162556	38159958	C	T	0.44	(732)	0.47	(829)	0.17	(147)	0.52	(438)	0.30	(256)	0.21	(190)	0.50	(449)	0.28	(251)
rs10175368	38161365	T	C	0.28	(471)	0.27	(478)	0.08	(65)	0.41	(341)	0.51	(431)	0.07	(62)	0.40	(354)	0.53	(472)

Allele 1 is the minor allele in each case. Note that SNPs are presented in genomic order and the gene is transcribed from the reverse strand.

Single SNP association analysis was conducted for five genetic models in Plink. No SNP was associated under the allelic test, nor in any of the other genetic models (Table 3). When the analysis was restricted to severe cases and elderly controls (≥81 years) only, SNPs rs162562 and rs10916 were nominally associated (p<0.05); however, these results do not survive correction for the number of SNPs assessed. The associations were also nominally significant under the recessive model and trend test. In addition, a logistic regression adjusted for age and sex was conducted. Nominally significant results at the same two SNPs were observed in the severe cohort, but do not survive multiple testing correction (Table 3).

**Table 3:** P-value for association of each SNP with POAG.

**All cases (n=860 versus all controls (n=897)**				** **	** **	** **
**SNP**	**Allelic**	**Genotypic**	**Dominant**	**Trend**	**Recessive**	Adjusted*
rs162549	0.637	0.725	0.853	0.643	0.424	0.822
rs10916	0.140	0.325	0.217	0.145	0.238	0.054
rs162562	0.112	0.268	0.185	0.116	0.120	**0.040**
rs1800440	0.617	0.614	0.848	0.620	0.325	0.259
rs1056836	0.976	0.711	0.674	0.977	0.600	0.430
rs162556	0.071	0.117	0.307	0.066	0.042	0.078
rs10175368	0.422	0.716	0.490	0.420	0.533	0.223
**Severe cases (n=396) versus Elderly controls (n=452)**				** **	** **	** **
**SNP**	**Allelic**	**Genotypic**	**Dominant**	**Trend**	**Recessive**	Adjusted*
rs162549	0.686	0.826	0.585	0.695	0.923	0.824
rs10916	**0.018**	0.060	**0.019**	**0.020**	0.280	**0.016**
rs162562	**0.019**	0.063	**0.020**	**0.022**	0.299	**0.017**
rs1800440	0.381	0.687	0.413	0.386	0.612	0.531
rs1056836	0.150	0.354	0.287	0.156	0.201	**0.040**
rs162556	0.112	0.040	0.772	0.108	0.013	0.129
rs10175368	0.307	0.502	0.481	0.309	0.268	0.091

Results are presented for 5 genetic models and the adjusted model for the full sample and the sample restricted to severe cases and elderly controls. Nominally significant p-values are highlighted in bold. The asterisk indicates p-values adjusted for age and sex in a logistic regression.

Haplotype analysis was conducted in Plink. No overall association between the *CYP1B1* locus and POAG was detected in either the full sample (p=0.140) nor the severe cases and elderly controls (p=0.189); however, one specific haplotype of the seven SNPs (AGCAGCC) was nominally associated in both data sets (Table 4). This is a relatively common haplotype that is slightly under-represented in POAG cases, particularly severe cases. The association does survive correction for multiple testing of the seven common haplotypes in the severe cases (corrected p-value=0.028). The associated haplotype is the most common haplotype to carry a C at SNP rs162562, which was nominally significant in the single SNP analysis of the severe cases. This allele is observed in only one other haplotype (AGCAGTG) which only differs from the associated haplotype at the 6th SNP (rs162556), but is rarer and is not associated with POAG.

**Table 4:** Association of common hapltoypes (>1% frequency).

** **	**Full sample (over-all p-value=0.156)**				**Restricted sample (over-all p-value=0.140)**			
**Haplotype**	**f cases**	**f controls**	**p-value**	**OR (95%CI)**	**f cases**	**f controls**	**p-value**	**OR (95%CI)**
ATAGCCC	0.19	0.18	0.536	1.06 (0.89–1.25)	0.20	0.18	0.305	1.13 (0.89–1.44)
ATAACTT	0.28	0.27	0.452	1.06 (0.91–1.23)	0.28	0.26	0.304	1.12 (0.90–1.39)
TTAAGTC	0.23	0.22	0.303	1.00 (0.93–1.28)	0.23	0.23	0.733	1.04 (0.83–1.30)
AGCAGCC	0.16	0.18	0.037	0.83 (0.69–0.90)	0.13	0.19	0.004*	0.68 (0.52–0.89)
TTAAGCC	0.02	0.01	0.256	1.09 (0.93–1.28)	0.01	0.01	0.617	1.26 (0.50–3.17)
AGCAGTC	0.05	0.04	0.441	1.14 (0.82–1.57)	0.05	0.04	0.351	1.23 (0.90–1.91)
ATAACCC	0.08	0.09	0.115	0.82 (0.64–1.05)	0.08	0.09	0.811	0.96 (0.68–1.36)

Results are presented for the full sample and the sample restricted to severe cases and elderly controls. All p-values and odds ratios (OR) are shown for the specific haplotype versus all other hapltoypes. Nominally significant values are in bold. The asterisk indicates survives Bonferonni correction for 7 observed haplotypes tested.

## Discussion

The association of the *CYP1B1* gene with PCG is well understood in populations world-wide, although the mechanism of disease is not. The gene is also associated with JOAG and may interact with the *MYOC* gene to cause the early onset observed in JOAG families. However, the role of *CYP1B1* in later onset POAG is unclear. The majority of studies to date have sequenced the coding region of the gene in a small cohort of POAG patients and most have identified missense mutations not observed in a control cohort. This approach has identified many apparent mutations that may contribute to the risk of POAG in rare cases, but does not provide evidence for a contribution of this locus to most (or even a significant proportion) of POAG cases. In addition, there are many reported missense, frameshift and truncating variants of this gene in non-POAG individuals, many of which have not been reported in the POAG cohorts, making interpretation of the published data in relation to POAG susceptibility difficult.

The present *CYP1B1* study is the largest cohort of POAG patients examined to date (n=860) and we were well powered to identify common genetic variants at the level of relative risk of 1.1 or 1.2. In addition, all controls (n=898) are at least 50 years of age and have been thoroughly examined for glaucoma phenotypes. We have taken a tag SNP approach to assess the role of common variation throughout the *CYP1B1* locus for an association with POAG in both a general POAG cohort as well as a cohort of severe (typically slightly younger onset) cases compared to elderly (>81 years) examined normal controls. These data do not provide evidence for a substantial role of this locus in POAG, although one haplotype may be protective for severe glaucoma . The odds ratio for the nominally associated haplotype is 0.68 when compared to all other common haplotypes. Power calculations [22] revealed that for a sample of this size (860 cases versus 898 controls) under an additive model we had 88% power to detect a genotype relative risk of 0.68 (or 1.47) for an allele frequency of 0.16 at α=0.007 (allowing for multiple testing of 7 haplotypes). Thus we are adequately powered to detect the effect size observed in this study.

The use of tag SNPs in case-control association studies is ideally suited to testing hypotheses of common variation causing a common disease. It will not detect individual rare variants occurring on multiple genetic backgrounds. Thus, this study does not rule out a role for *CYP1B1* in POAG, but does indicate that common variation in the gene (including common coding SNPs L432V and N453S) is not associated with POAG in general, but may be associated with severe POAG in a Caucasian population.
